# Mesenchymal Stromal Cell Therapy Reverses Detrusor Hypoactivity in a Chronic Kidney Patient

**DOI:** 10.3390/biomedicines11010218

**Published:** 2023-01-14

**Authors:** Henrique Rodrigues Scherer Coelho, Silvia Cordeiro das Neves, Jovino Nogueira da Silva Menezes, Andréia Conceição Milan Brochado Antoniolli-Silva, Rodrigo Juliano Oliveira

**Affiliations:** 1 Center for Studies in Stem Cells, Cell Therapy and Genetic Toxicology (CeTroGen), Faculty of Medicine Dr. Hélio Mandetta (FAMED), Federal University of Mato Grosso do Sul (UFMS), Campo Grande 79070-900, Mato Grosso do Sul, Brazil; 2 Graduate Program in Health and Development of the Midwest Region, Faculty of Medicine Dr. Hélio Mandetta (FAMED), Federal University of Mato Grosso do Sul (UFMS), Campo Grande 79070-900, Mato Grosso do Sul, Brazil; 3 Clínica Samari, Campo Grande 79020-011, Mato Grosso do Sul, Brazil

**Keywords:** urinary bladder, urodynamics, chronic renal failure, lower urinary tract, case report

## Abstract

Detrusor hypoactivity (DH) is characterized by low detrusor pressure or a short contraction associated with low urinary flow. This condition can progress to chronic renal failure (CRF) and result in the need for dialysis. The present case report demonstrates that a patient diagnosed with DH and CRF who received two transplants with 2 × 10^6^ autologous mesenchymal stromal cells at an interval of 30 days recovered the contractile strength of the bladder and normalized his renal function. The patient had a score of 19 on the ICIQ-SF before cell therapy, and that score was reduced to 1 after transplantation. These results demonstrate that there was an improvement in his voiding function, urinary stream and urine volume as evaluated by urofluxometry. In addition, a urodynamic study carried out after treatment showed an increase in the maximum flow from 2 mL/s to 23 mL/s, the detrusor pressure in the maximum flow from 21 cm H_2_O to 46 cm H_2_O and a BCI that went from 31 to 161, characterizing good detrusor contraction. Thus, in the present case, the transplantation of autologous mesenchymal stromal cells proved to be a viable therapeutic option to allow the patient to recover the contractile strength of the bladder, and reversed the CRF.

## 1. Introduction

Detrusive hypoactivity (DH) is characterized by low detrusor pressure or a short contraction associated with low urine flow. The result is prolonged emptying of the bladder and/or failure to achieve complete emptying [1].

DH patients develop signs and symptoms that can worsen the condition. Among these, there is a high postvoiding residue that leads to an increase in recurrent urinary infections, the formation of urinary tract stones and even acute renal failure [2,3]. To date, there is no effective treatment to restore the function of bladder contractility. In view of the above, the present research reports a case of a patient with DH and chronic renal failure (CRF) treated with autologous mesenchymal stromal cells (AMSCs).

A 54-year-old male patient with no history of smoking or alcohol use, systemic arterial hypertension, a history of acute urinary retention for 18 months and acute renal failure was referred to the Urology Service of Maria Aparecida Pedrossian University Hospital (HUMAP). The patient was previously monitored by the nephrology service at the same hospital and was on hemodialysis for 6 months. The patient had also undergone endoscopic prostate resection to treat prostate hypertrophy. The anatomopathological examination resulted in a diagnosis of benign prostatic hyperplasia. After surgical treatment, the patient still complained of a weak and intermittent urinary stream, a feeling of incomplete emptying, terminal drip and urinary loss.

The patient underwent bladder catheterization, hematological and biochemical tests, including presurgical tests, ultrasound of the urinary and prostate apparatus, urethrocystoscopy (30° optics, 19 fr cystoscope, attached to a Storz camera) and a urodynamic study consisting of initial urofluxometry, differential cystometry and a pressure flow study performed on Dynapack MPX 816 urodynamic equipment.

To evaluate his quality of life, the validated instrument “International Continence on Incontinenca Questionnaire” was used in Short Form (ICIQ-SF). The data collection instrument was applied at the beginning of the research and 60 days after the last transplant.

The patient was offered treatment with mesenchymal stem cells (CEP/CONEP number # 2,745,746). After accepting the intervention and signing the informed consent form, the patient was subjected to the collection of peripheral fat from the inside of the right and left thighs through liposuction. The procedure was performed by a plastic surgeon on an outpatient basis. Asepsis was performed with 4% aqueous chlorhexidine, and sterile fields were placed. Then, anesthesia was performed with 125 mL of anesthetic solution (lidocaine 20% without adrenaline, 0.9% saline and 8.4% sodium bicarbonate) to promote tissue lipodistension. A total of 200 milliliters of the solution was removed with the aid of a liposuction cannula (3 mm). The material was stored in a sterile flask containing phosphate buffer solution (PBS) with 1% antibiotic/antimycotic (penicillin, streptomycin, amphotericin B, Sigma-A5955, lot 097 M4875 V). The material was transported to the Center for Studies on Stem Cells, Cell Therapy and Toxicological Genetics (CeTroGen/HUMAP) and processed according to a conventional routine for the extraction and cultivation of AMSCs [4]. In short, adipose tissue was performed by enzymatic digestion with Collagenase type 1 (Gibco^®^; Nº 9001-12-1; 290 U/mg) as described. For every 1 mL of fat, 3 mL of collagenase was used. The culture medium used was low glucose Dulbecco’s modified Eagle’s medium (DMEM) (Sigma^®^) with 2.5 g of Free Acid HEPES (Sigma^®^), 3.7 g of sodium bicarbonate (Dinâmica^®^) and 10% of bovine fetal serum (BFS) (Gibco^®^, Sigma-Aldrich^®^). The fractionated vascular stromal pellet acquired at the end of the process was resuspended in 5 mL of DMEM and seeded in a culture 25 cm^3^ flask. After 3 days, the culture was washed three times with 3 mL PBS to eliminate non-adherent cells and debris. DMEM culture medium, 10% BFS and 1% antibiotic (Penicilina/Estreptomicina/Amphotericin, Sigma-Aldrich^®^), was changed every 72 h (5 mL). Upon reaching 70–80% confluence in the flasks, they were trypsinized (0.25% trypsin/3 min), divided and seeded in new larger flasks (75 cm^2^).

A separate aliquot of cells was cultured for immunophenotyping by flow cytometry (CD105, CD90, CD34 and CD133) and cell differentiation (adipogenic, osteogenic and chondrogenic) [4].

For the preparation of the biological material for cell therapy, the cells were trypsinized and washed thoroughly, using a procedure adapted from Schweich–Adami [4]. For the syringe preparation, the AMSC vials were first washed with 3 mL of PBS (three times), then they were trypsinized (0.25% trypsin/3 min) and the contents were centrifuged (1200 rpm/5 min) in a falcon tube. The supernatant was then discarded and the cell pellet was resuspended in 3 mL PBS. Afterward, falcon tubes were centrifuged again (1200 rpm/5 min). This process was repeated three times [4].

All biological materials used for transplantation were previously submitted for cell viability analysis, using trypan blue, and conventional microbiological routine (aerobiosis and microaerophilia), for 72 h of incubation [4].

After 60 days, the patient underwent urethrocystoscopy on an outpatient basis for transplantation of 2 × 10^6^ AMSCs. The transplant was performed by intravesical injection with a cystoscopic needle (20 gauge) at points in the body of the bladder above the bladder trigone. A second transplant was performed 30 days after the first following the same procedure.

The patient was followed up clinically during and after the transplant to check for possible complications. The patient also underwent urine culture tests.

## 2. Results

The patient underwent a delayed bladder catheter that drained 1200 mL of urine.

On physical examination, no visceromegaly or lymphonomegaly was noted. Upon urological examination, normal external genitalia were noted. The abdomen was bulging and there was pain in the hypogastric region. Digital rectal examination revealed a 40 g prostate, without nodules and painless. The blood count and the coagulogram were normal. Biochemical tests indicated incipient chronic kidney disease with urea of 59 mg/dL and creatinine of 1.8 mg/dL. Ultrasound of the urinary tract showed an effort bladder and signs of chronic bilateral nephropathy. Prostate ultrasound showed a 45 g prostate and a marked postvoiding residue.

Urethrocystoscopy was normal. The penile, bulbar and membranous urethra were patent. The prostatic urethra showed signs of previous endoscopic resection and an open bladder neck. The ureteral meatus was topical and there were trabeculated bladder walls.

Subsequently, the patient underwent a urodynamic study to assess his voiding dysfunction. The initial urofluxometry showed a maximum flow of 4 mL/s, an average flow of 1 mL/s, a urine volume of 19 mL and a postvoiding residue of 800 mL. Differential cystometry showed a maximum cystometric capacity (CCM) of 550 mL, a bladder compliance of 45 mL/cmH_2_O, decreased sensitivity, no losses with effort maneuvers and an absence of uninhibited contraction of the detrusor. The flow pressure study showed a maximum flow (Qmax) of 2 mL/s and detrusor pressure at a maximum flow (PdetQmax) of 21 cmH_2_O, a urine volume of 82 mL, and a detrusor contractility index (bladder contractility index: BCI = PdetQmax + 5 × Qmax) of 31. The values obtained with this urodynamic study were compatible with the diagnosis of detrusor hypoactivity (Table 1).

The standard treatment for DH was instituted with clean intermittent catheterization with a maximum volume in each 400 mL drainage and periodic monitoring with a voiding diary.

**Figure 1:**
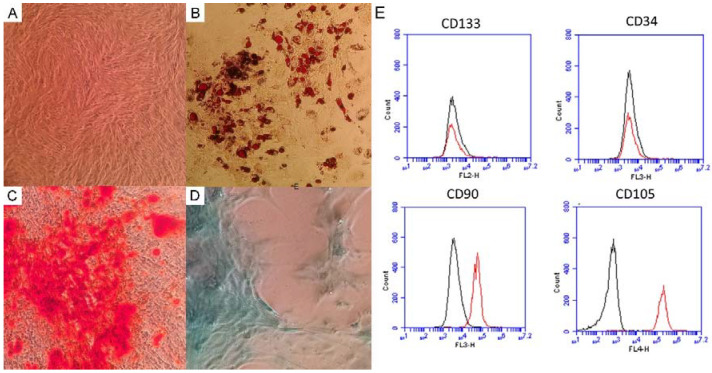
Morphology, characterization and differentiation potential of adipose-derived stromal cells–(**A**) Undifferentiated culture demonstrating cells with fibroblast characteristics, (**B**) adipogenic differentiation culture and lipid vacuoles stained with Oil Red O, (**C**) osteogenic differentiation culture and calcium deposits stained with Alizarin Red and (**D**) chondrogenicdifferentiation culture and glycosaminoglycan-rich extracellular matrix stained with Alcian Blue. (**E**) Immunophenotypic profile of mesenchymal stromal cells. Cells expressed the CD 90 and CD105 markers and did not express CD133 and CD34.

The immunophenotypic profile of AMSCs expressed the CD90 and CD105 markers and it did not express CD133 and CD34. So, this result corroborated the fact that these are mesenchymal stromal cells (Figure 1E). The differentiation was confirmed by the presence of lipid droplets and an extracellular matrix rich in calcium and glycosaminoglics after staining with Oil Red, Alizarida Red and Alcian Blue, respectively (Figure 1A–D), the second protocol of SCHWEICH [5].

The patient was followed up clinically during and after the transplant to check for possible complications, and none were observed. Uroculture exams did not indicate any infection.

A total of 60 days after completion of the transplant, the patient underwent a new urodynamic study. The initial urofluxometry showed a maximum flow of 21 mL/s, an average flow of 6 mL/s, a urine volume of 232 mL and a residue of 20 mL. Differential cystometry showed a maximum cystometric capacity of 450 mL, bladder compliance of 50 mL/H_2_O, decreased sensitivity with no loss or involuntary detrusor contraction. The flow-pressure study showed a maximum flow of 23 mL/s and a PdetQmax of 46 cm H_2_O. The urine volume was 537 mL, and the BCI reached 161.

The score on the ICIQ-SF questionnaire was only one.

The patient was followed up for 6 months, and his condition of chronic renal failure was reversed. This fact guaranteed the patient an improvement in quality of life and his withdrawal from hemodialysis, which was performed three times a week before the transplant.

## 3. Discussion

DH is a voiding dysfunction characterized by low urinary flow, decreased detrusor pressure or a poor contraction duration that leads to incomplete emptying of the bladder [1,6]. The diagnosis requires a clinical evaluation, ultrasound of the urinary tract and urethrocystoscopy for the proper differential diagnosis of the causes of the symptoms of the lower urinary tract. The gold standard for confirming this diagnosis is a pressure flow study carried out during a urodynamic study, showing a maximum flow < 15 mL/s and Pdet < 30 cm H_2_O [1,7].

The patient had a medical age compatible with a diagnosis of benign prostatic hypertrophy (BPH), as up to 50% of patients over 50 years of age can develop this disease, and up to 40% of men under 65 years of age may present with detrusor hypoactivity [8,9].

The clinical history presented represents the expected evolution of patients who have BPH and have a late diagnosis and treatment. The patient developed chronic urinary retention, requiring the use of a Foley catheter, and then went on to develop chronic renal failure. Even after surgical treatment of the BPH through endoscopic prostate resection, the patient had signs of chronic urinary retention with a palpable bladder globe, elevated postvoiding residue, and a weak, intermittent and prolonged urinary jet. This condition has also been reported by Aldamanhori, Chapple [2]. These conditions had a negative impact on the patient’s quality of life, as evidenced by his score of 19 on the ICIQ-SF, which has a maximum score of 21 [10].

Ultrasonography showed signs of chronic bilateral nephropathy, a thickened bladder and elevated postvoiding residue. Urethrocystoscopy demonstrated that there was no obstruction of the lower urinary tract due to urethral stenosis and that the bladder neck was open. These facts together suggest DH [9]. The urodynamic study demonstrated a maximum flow of 2 mL/s and a PdetQmax of 21 cm H_2_O. The calculated BCI was 31, well below 100, which confirms the diagnosis of DH [1,11].

The patient was treated with conservative measures such as clean intermittent catheterization and surveillance for kidney function and urinary tract infections [12]. However, these interventions were not efficient, and the patient’s general condition would certainly have evolved into complications due to chronic renal failure requiring hemodialysis throughout his life. Chronic kidney patients generate high costs for the Unified Health System (SUS) in addition to having a poor quality of life and low survival rate [13,14].

In this context of the lack of satisfactory results with conventional therapies, therapy with AMSCs proved to be efficient in reversing the condition of chronic renal failure and improving the patient’s quality of life. Such improvements are represented by a reduction of 18 points on his ICIQ-SF score, which is an internationally validated questionnaire that is widely used in this field [15,16].

The improvement in the quality of life of the patient and in his renal functions also supports the improvement in voiding function, urinary stream and urine volume evaluated by the urofluxometry exam. In addition, the urodynamic study carried out after treatment showed an increase in the maximum flow of 2 mL/s to 23 mL/s, the detrusor pressure in the maximum flow from 21 cm H_2_O to 46 cm H_2_O and a BCI that went from 31 to 161, characterizing a good detrusor contraction [1,12]. These values removed the patient from the diagnosis of DH and demonstrated a reversal of the chronic renal failure being caused by the DH. The patient also reported satisfaction with the voiding function and the emptying capacity of the bladder since the residual volume before transplantation of the AMSCs was 800 mL and it is currently less than 20 mL during clean intermittent catheterization, a volume that is negligible and well within the parameters considered normal [17].

This case report is unprecedented. In the research literature, no other study was found that demonstrated the effects of adipose-tissue-derived multipotent mesenchymal stromal cells in the treatment of chronic renal failure associated with DH. According to Dominici et al. [18] and Mallis et al. [19], multipotent mesenchymal stromal cells are plastic adherent when maintained in standard culture conditions; must express CD105, CD73 and CD90 and lack expression of CD45, CD34, CD14 or CD11b, CD79a or CD19 and HLA-DR surface molecules; and must differentiate to osteoblasts, adipocytes and chondroblasts in vitro.

Historically, mesenchymal stromal cells were first described by Friendenstein as hematopoietic cells that would support the production of bone marrow [20,21,22]. The evolution of studies later proposed the existence of a stromal stem cell to maintain the microenvironment of the bone marrow and a hematopoietic stem cell to maintain hematopoiesis [22,23]. Cells similar to those responsible for the bone marrow microenvironment have also been found in other tissues. Therefore, currently, mesenchymal stromal cells are considered as a set of heterogeneous cells with a self-renewal capacity [19].

## 4. Conclusions

In conclusion, we infer that AMSC transplantation, in the present case, proved to be a viable therapeutic option since the patient recovered bladder contractile strength and thus reversed his chronic renal failure.

## Figures and Tables

**Table 1:** Absolute values and percentage variation of the results of urofluxometry, cystometry and study of pressure flow and score of the International Continence on Incontinence Questionaire-Short Form, before and after cell therapy.

Urofluxometry			
**Parameters**	Before	After	%
Maximum Flow	4	21	425
Average Flow	1	6	500
Urinary volume	19	232	1121
Residue	800	20	−98
Cystometry			
**Parameters**	Before	After	%
CCM	500	405	−19
Urinary Loss	yes	no	
Hyperactivity	no	no	
Complacency	45	50	11
Pressure Flow Study			
**Parameters**	Before	After	%
Maximum Flow	2	23	1050
Maximum Detrusion Pressure	21	46	119
Urinary volume	82	537	555
BCI	31	161	419
International Continence on Incontinenca Questionaire-Short Form			
**Parameters**	Before	After	%
ICIQ-SF	19	1	−95%

Legend: CCM—Maximum cystometric capacity; BCI—Bladder Contractility Index.

## Data Availability

All data generated or analysed during this study are included in this published article.
